# Identification of novel immune subtypes and potential hub genes of patients with psoriasis

**DOI:** 10.1186/s12967-023-03923-z

**Published:** 2023-03-08

**Authors:** Yingxi Li, Lin Li, Yao Tian, Jing Luo, Junkai Huang, Litao Zhang, Junling Zhang, Xiaoxia Li, Lizhi Hu

**Affiliations:** 1 Immunology Department, Key Laboratory of Immune Microenvironment and Disease (Ministry of Education), Tianjin Medical University, Tianjin, 300070 China; 2 Department of Dermatology, Tianjin Academy of Traditional Chinese Medicine Affiliated Hospital, Tianjin, 300120 China; 3 Department of General Surgery, Tianjin Medical University General Hospital, Tianjin, 300052 China

**Keywords:** Psoriasis, Immune landscape, Hub genes, Immune subtypes, Bioinformatic analysis

## Abstract

**Background:**

Psoriasis is a common, chronic and relapsing immune-related inflammatory dermal disease. Patients with psoriasis suffering from the recurrences is mainly caused by immune response disorder. Thus, our study is aimed to identify novel immune subtypes and select targeted drugs for the precision therapy in different subtypes of psoriasis.

**Methods:**

Differentially expressed genes of psoriasis were identified from the Gene Expression Omnibus database. Functional and disease enrichment were performed by Gene Set Enrichment Analysis and Disease Ontology Semantic and Enrichment analysis. Hub genes of psoriasis were selected from protein–protein interaction networks using Metascape database. The expression of hub genes was validated in human psoriasis samples by RT-qPCR and immunohistochemistry. Further, novel immune subtypes of psoriasis were identified by ConsensusClusterPlus package and its association with hub genes were calculated. Immune infiltration analysis was performed, and its candidate drugs were evaluated by Connectivity Map analysis.

**Results:**

182 differentially expressed genes of psoriasis were identified from GSE14905 cohort, in which 99 genes were significantly up-regulated and 83 genes were down-regulated. We then conducted functional and disease enrichment in up-regulated genes of psoriasis. Five potential hub genes of psoriasis were obtained, including SOD2, PGD, PPIF, GYS1 and AHCY. The high expression of hub genes was validated in human psoriasis samples. Notably, two novel immune subtypes of psoriasis were determined and defined as C1 and C2. Bioinformatic analysis showed C1 and C2 had different enrichment in immune cells. Further, candidate drugs and mechanism of action that applicable to different subtypes were evaluated.

**Conclusions:**

Our study identified two novel immune subtypes and five potential hub genes of psoriasis. These findings might give insight into the pathogenesis of psoriasis and provide effective immunotherapy regimens for the precise treatment of psoriasis.

**Supplementary Information:**

The online version contains supplementary material available at 10.1186/s12967-023-03923-z.

## Introduction

Psoriasis is a common, chronic and relapsing immune-related dermal disease, which is prevalent in 2–4% of populations worldwide. The underlying pathogenesis of psoriasis raised from an interworking among immune, heredity and environmental factors, such as trauma, drugs, infections, smoking, alcohol and stress. In terms of the molecular mechanism of psoriasis, the excessive proliferation of keratinocyte and its pathogenesis contains the dysfunction of immune system. To be specific, the uncontrolled immune response is mediated by T lymphocytes and various immune cells, thus leading to the phenotypical manifestations in psoriasis [1, 2].

In recent years, biological agents (e.g. TNFα inhibitors, IL-23 inhibitors, IL-17 inhibitors, and IL-12/23 inhibitors) have become important treatments for patients with moderate to severe psoriasis, which block the proinflammatory cytokines and lymphocyte activation in psoriasis [3–6]. Moreover, researchers found that biological agents can effectively alleviate the clinical symptom and reduce both the psoriasis area and severity index score in patients [7–9]. In spite of significant advances in unravelling the pathogenesis of psoriasis and the success in therapeutic interventions, patients still suffer from the frequent adverse events and recurrences [7, 10, 11]. Thus, there is an urgent need to provide potential biomarkers and identify novel immune subtypes of psoriasis, thus selecting targeted drugs for the precision therapy in psoriasis.

In this study, differentially expressed genes (DEGs) in psoriasis were identified from gene expression profile (GSE14905) in Gene Expression Omnibus (GEO) database. To analyze the functional enrichment and disease enrichment in up-regulated genes of psoriasis, Gene Set Enrichment Analysis, Kyoto Encyclopedia of Genes and Genomes (KEGG), Disease Ontology Semantic and Enrichment (DOSE) analysis were performed. Then, 5 potential hub genes (SOD2, PGD, PPIF, GYS1, AHCY) of psoriasis were identified by protein–protein interaction (PPI) networks using Metascape database. Further, the expression of hub genes was verified in human psoriasis samples in vitro*.* In addition, the correlation between hub genes and immune infiltrating cells collected from public database was calculated using Pearson correlation. We found a significant association between the higher expression levels of AHCY and macrophages, PPIF and activated CD8^+^ T cell, PGD and activated dendritic cell, respectively. These cells play an important role in the pathogenesis of psoriasis. As we known, many investigators have now demonstrated that multiple immune cell types are present in psoriasis, including dendritic cells, T cells and macrophages, which contribute to disease pathogenesis and drive keratinocyte proliferation [12]. Notably, in order to provide effective therapeutic applications for the precise treatments of psoriasis, novel immune subtypes of psoriasis were determined using ConsensusClusterPlus package. Based on these clusters, the immune infiltration landscape was further identified and its correlation with candidate drugs were evaluated for different immune subtypes of psoriasis.

## Materials and methods

### Identification of DEGs

GEO (https://www.ncbi.nlm.nih.gov/geo/) is a public database containing amount of high-throughput gene expression and genomic hybridization experiments [13]. In order to explore the differentially expressed genes of psoriasis, GSE14905 was selected via the “limma” package in R studio software 4.1.1 (https://www.rstudio.com/), |logFC|≥ 1 and adjusted *P* value < 0.05 were set as filter values. GSE14905 dataset includes skin biopsy samples from 21 normal healthy donors, 56 skin biopsy samples from 28 psoriasis patients who had matched lesioned and non-lesioned tissues, and 5 samples from psoriasis patients who only provided lesioned skin biopsies. Therefore, 33 lesioned psoriasis samples and 28 non-lesioned skin samples were conducted to perform the subsequent analysis in the study.

### Functional enrichment analysis

To analyze the molecular pathways involved in up-regulated genes of psoriasis, we performed GSEA and KEGG enrichment analysis using Metascape. Metascape (http://metascape.org/) is a web-based portal designed to provide a comprehensive gene list annotation and analysis resource for experimental biologists. In terms of design features, Metascape combines functional enrichment, interactome analysis, gene annotation, and membership search to leverage over 40 independent knowledge bases within one integrated portal [14]. Metascape was also conducted to perform TRRUST analysis and PPI networks. TRRUST (http://www.grnpedia.org/trrust) contains 8444 and 6552 transcription factors (TFs) targeted regulatory relationships of 800 human TFs and 828 mouse TFs, respectively.

### Screening hub genes and correlation analysis

To identify hub genes, PPI networks were performed based on up-regulated genes of psoriasis using Metascape database. PPI network is composed of individual proteins that interact with each other to participate in several biological processes. Protein–protein interaction enrichment analysis has been carried out by with the following databases: STRING, BioGrid, OmniPath, InWeb_IM. If the network contains between 3 and 500 proteins, the Molecular Complex Detection (MCODE) algorithm has been applied to identify densely connected network components.

Further, the correlation between hub genes and immune infiltration cells was calculated using Pearson correlation analysis, and the results were visualized using the R package. *P*-value < 0.05 was considered statistically significant.

### Identification of immune subtypes and immune infiltration analysis in psoriasis

To identify immune subtypes of psoriasis, we firstly collected 1793 immune-related genes from IMMPORT database (https://www.immport.org/home). Then, 13 genes were obtained by intersection with 1793 immune-related genes and 99 up-regulated genes of psoriasis. These 13 genes were further used to construct consensus clustering and identify immune subtypes of psoriasis using ConsensusClusterPlus package [15]. The partition around medoids (PAM) algorithm was used with distance quantified as ‘1—Pearson’ correlation coefficient [16]. 100 replicates of bootstraps were carried out, which included 80% patients of the GEO cohort. The K value of cluster was varied from 2 to 4 and the optimal value was depending on the consensus matrix and the consensus cumulative distribution function. Then, the correlation between hub genes and 13 genes was calculated using Pearson correlation analysis. Dimension reduction was performed by Rtsne package.

To assess the immune infiltration profile in psoriasis, GSEA algorithm was performed with 28 types of immune infiltrating cells in C1 and C2 psoriasis samples. Differences were calculated using the Wilcoxon test, and *p*-value < 0.05 was considered to indicate statistically significant results. Based on the results of immune infiltration analysis, the correlation between 13 genes involved in immune subtypes of psoriasis and 782 genes involved in biomarkers of 28 immune infiltrating cells was analyzed to clarify the relationship between immune characteristics and psoriasis-related genes. The correlation matrix was visualized using Pearson correlation analysis.

### Connectivity map analysis

To investigate the candidate drugs for different subtypes of psoriasis, Connectivity Map analysis was performed (https://clue.io/). The Connectivity Map is a pharmacogenome-based tool, which is conducted to explore the potential candidates targeting immunophenotype-related biological pathways and genes as well as understanding the potential MoAs of drugs [17]. To be specific, 300 genes containing top 150 up-regulated genes and top 150 down-regulated genes were obtained from difference analysis between C1 and C2 subtypes of psoriasis. Then, these 300 genes were used in the analysis of Connectivity Map.

### RT-qPCR

Total RNA was prepared from cells using TRIzol Reagent (Invitrogen, Carlsbad, CA, USA), and 1 μg of total RNA was used for complementary DNA synthesis with a Quantscript Reverse Transcription Kit (TransGen Biotech, Beijing, China). Real-time polymerase chain reaction (PCR) reactions were performed using SYBR Green PCR Master Mix (TransGen Biotech, Beijing, China) on the 7500 Real-Time PCR System (Applied Biosystems, Waltham, MA, USA). The sequences of primers are shown in Additional file 1: Table S1.

### Hematoxylin–eosin staining and immunohistochemistry

For hematoxylin–eosin (HE) staining, tissues were embedded in paraffin and sectioned into 4-μm intervals (Leica). For immunohistochemistry (IHC), tissue sections were deparaffinized, rehydrated, and permeated using Triton X 100 (T8200, Solarbio, Beijing, China) and followed by antigen retrieval using EDTA Antigen Retrieval solution (c1034, Solarbio, Beijing, China). The sections were incubated with Anti-SOD2 antibody (bs-23402R, Bioss, Shanghai, China), Anti-PPIF antibody (bs-7624R, Bioss), Anti-GYS1 antibody (10566-1-AP, Proteintech, Wuhan, China), Anti-AHCY antibody (DF7260, Affinity, Beijing, China) and Anti-PGD antibody (14718-1-AP, Proteintech) at 4 °C overnight followed by a biotinylated secondary antibody (diluted at 1:200) at RT for 60 min. Then, the sections were stained with DAB staining solution (AR1022, BOSTER Biological Technology, Wuhan, China) and counterstained with hematoxylin.

### Statistical analyses

Statistical analyses of RT-qPCR were presented as the mean ± standard deviation for at least three individual experiments and the statistical significance of differences was determined with the unpaired, two-tailed Student t test. (*P < 0.05; **P < 0.01; ***P < 0.001). In addition, other statistical analyses in this study were performed by R studio software 4.1.1.

## Results

### Identification of DEGs between lesioned psoriasis and non-lesioned skin samples

**Fig. 1:**
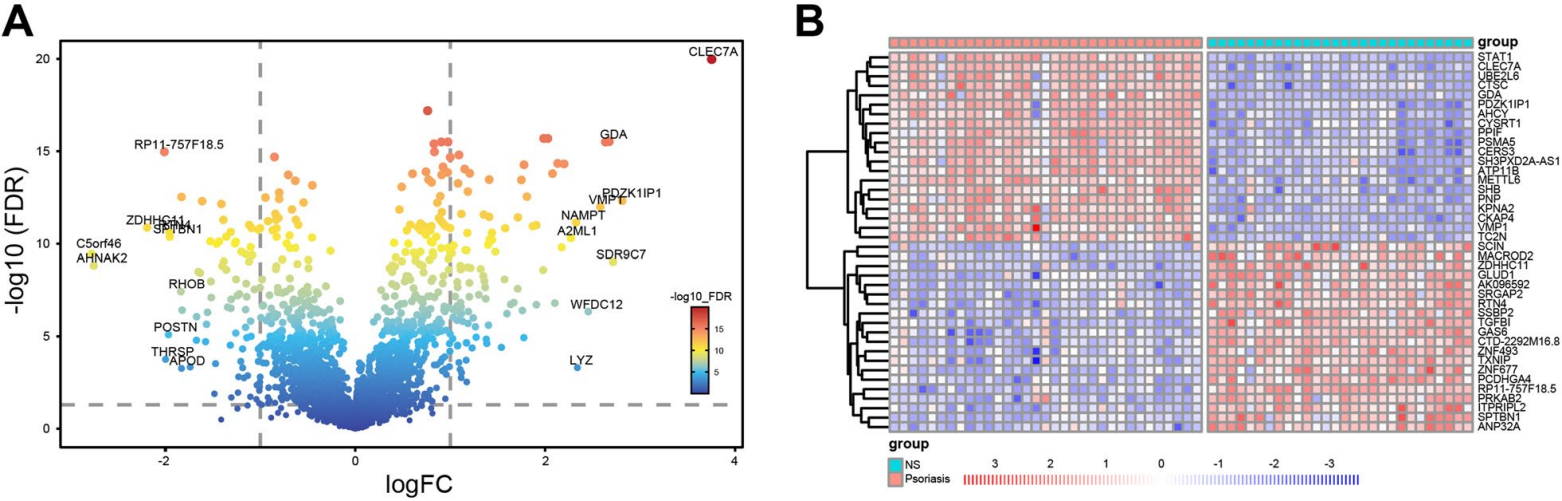
The DEGs from lesioned psoriasis samples and non-lesioned skin samples represented as volcano plots (**A**) and heatmaps (**B**)

Firstly, the flow chart of the research design was represented in Additional file 2: Fig. S1. To explore the DEGs from 33 lesioned psoriasis samples and 28 non-lesioned skin samples of patients, RNA-seq data in GSE14905 was analyzed and represented as volcano plots (Fig. 1A) and heatmaps (Fig. 1B). The results showed that 99 genes were significantly up-regulated, and 83 genes were down-regulated in patients with psoriasis.

### Functional enrichment analysis in psoriasis

**Fig. 2:**
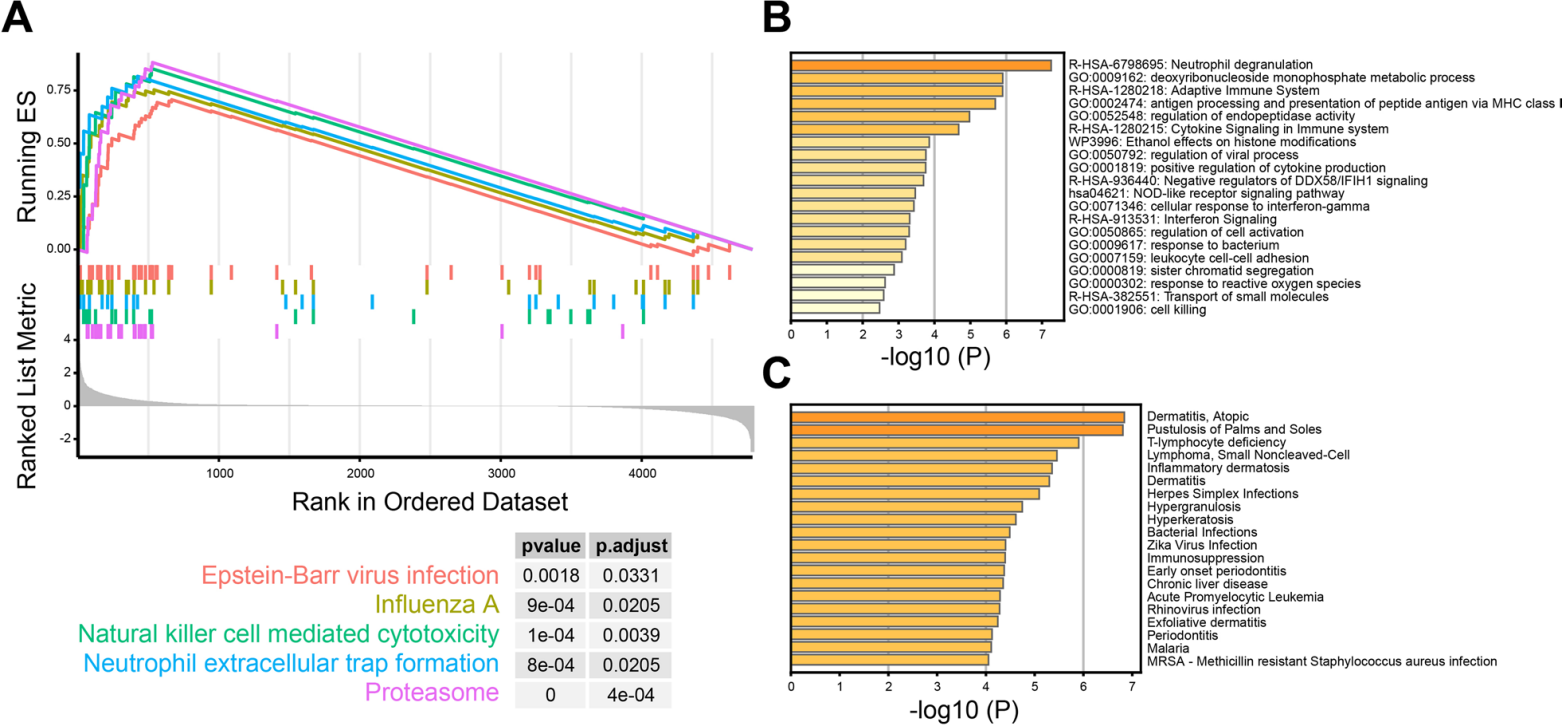
Functional enrichment analysis based on up-regulated genes of psoriasis. The molecular pathway enrichment by GSEA (**A**) and KEGG (**B**). **C** The disease enrichment by DOSE analysis

In order to analyze the molecular pathways and diseases involved in 99 up-regulated genes of psoriasis, GSEA analysis found that these genes were enriched in the Epstein-Barr virus infection (*P* = 0.0018) and neutrophil extracellular trap formation (*P* = 8e−04) (Fig. 2A). Simultaneously, KEGG analysis indicated the significant enrichment pathways, such as R-HSA-6798695: (neutrophil degranulation), R-HSA-1280218: (adaptive immune system) and GO:0002474: (antigen processing and presentation of peptide antigen via MHC class I) (Fig. 2B). In terms of Disease Ontology Semantic and Enrichment (DOSE) analysis, these 99 up-regulated genes were involved in immune related diseases, such as atopic dermatitis, pustulosis of palms and soles lymphoma, lymphoma and inflammatory dermatosis (Fig. 2C).

### Selection and analysis of Hub genes

**Fig. 3:**
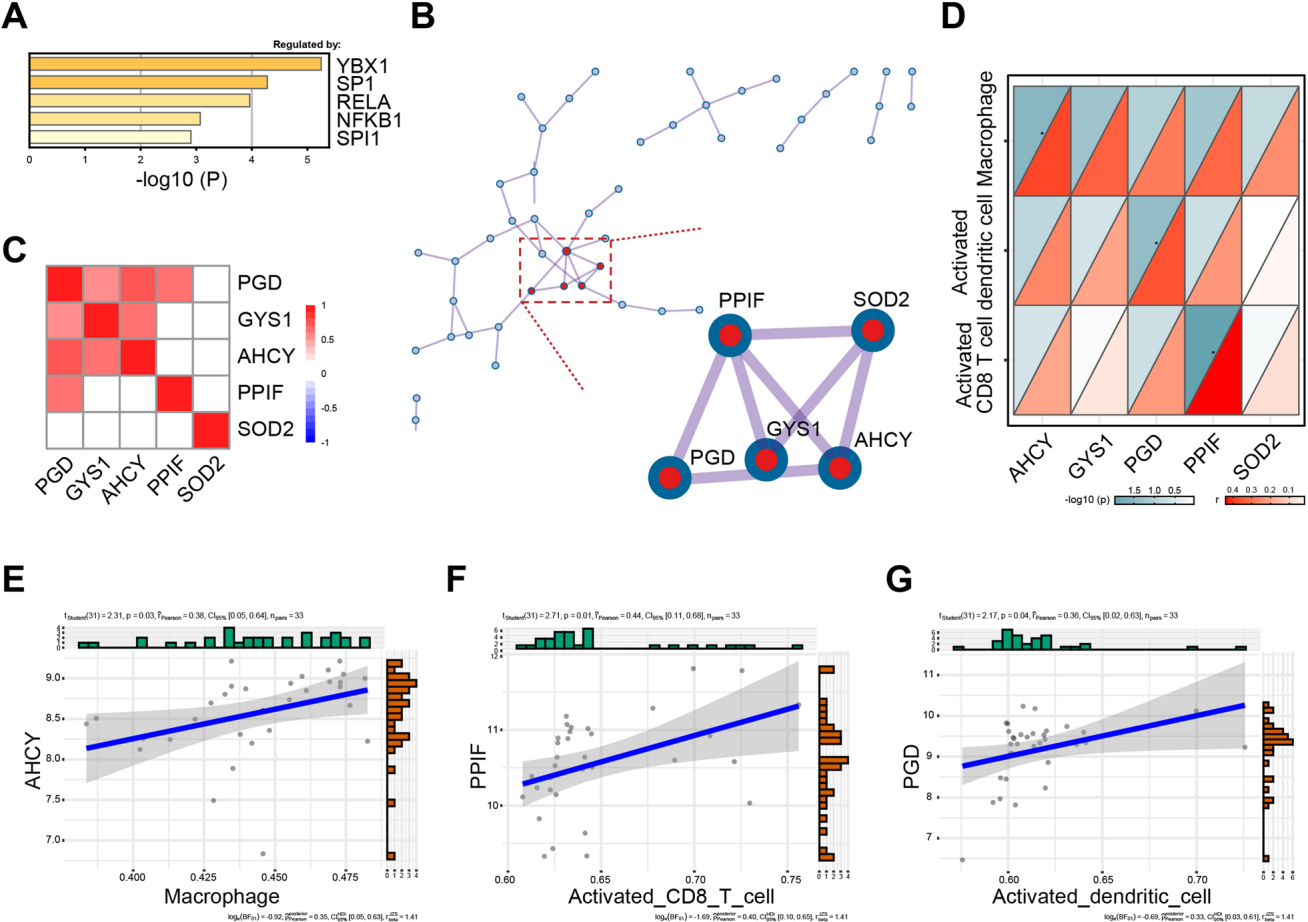
Identification of potential hub genes and its regulations. **A** Predicted potential upstream regulatory transcription factors of up-regulated genes in psoriasis. **B** PPI networks of 99 up-regulated genes in psoriasis. **C** The correlations of 5 hub genes by Pearson’s analysis. **D**–**G** Correlation of 5 hub genes expression with immune infiltration levels in psoriasis (**D**). Association of AHCY (**E**), PPIF (**F**) and PGD (**G**) expression with the number of infiltrating cells in psoriasis

As we known, TFs are important to regulate the expression of genes. According to the molecular mechanisms of these 99 genes regulation in psoriasis, the upstream transcription factors (TFs) of genes were firstly predicted using TRRUST analysis: YBX1, SP1, RELA, NFKB1 and SPI1 (Fig. 3A). To further explore the biological functions of up-regulated genes in psoriasis, PPI networks were performed using Metascape database (https://metascape.org/gp/index.html#/main/step1) (Fig. 3B). PPI networks selected five hub genes, which have pivotal roles in psoriasis: peptidylprolyl isomerase F (PPIF), superoxide dismutase 2 (SOD2), glycogen synthase 1 (GYS1), adenosylhomocysteinase (AHCY) and phosphogluconate dehydrogenase (PGD) (The details of the hub genes were represented in Table 1). As is shown in Fig. 3B, these 5 hub genes have connections with at least 3 genes and are more central than other genes. Further, we found the role of these five hub genes in skin inflammatory diseases. For example, researchers found the expression of SOD2 was elevated in skin lesion [18]. In terms of psoriasis, SOD2 functioned as a regulator in psoriatic macrophages, resulting in enhanced presence of mitochondrial ROS [19]. PGD, which is synthesized primarily in human skin, is mostly investigated in the context of allergic responses, particularly in atopic dermatitis lesions [20, 21]^.^ However, the potential role of PGD, PPIF, GYS1 and AHCY has not been explored in psoriasis. Therefore, the correlations of them were calculated by Pearson’s analysis, which showed significantly positive correlations among PGD, PPIF, GYS1 and AHCY (Fig. 3C). To further explore the relations between 5 hub genes and immune infiltration (Fig. 3D), we found a significant association between the higher expression levels of AHCY (Fig. 3E), PPIF (Fig. 3F) and PGD (Fig. 3G) and the increased immune infiltration of cells, such as macrophages, activated CD8^+^ T cell and activated dendritic cell, respectively.

**Table 1:** The details of hub genes

No.	Gene	Full name	Function
1	PPIF	Peptidylprolyl isomerase F	A member of the peptidyl-prolyl cis–trans isomerase family, which is part of the mitochondrial permeability transition pore in the inner mitochondrial membrane. Activation of this pore is thought to be involved in the induction of apoptotic and necrotic cell death
2	SOD2	Superoxide dismutase 2	A member of the iron/manganese superoxide dismutase family. It encodes a mitochondrial protein, which binds to the superoxide byproducts of oxidative phosphorylation and converts them to hydrogen peroxide and diatomic oxygen
3	GYS1	Glycogen synthase 1	Catalyzes the addition of glucose monomers to the growing glycogen molecule through the formation of α-1,4-glycoside linkages
4	AHCY	Adenosylhomocysteinase	Catalyzes the hydrolysis of S-adenosyl-L-homocysteine to form adenosine and homocysteine
5	PGD	Phosphogluconate dehydrogenase	Catalyzes the oxidative decarboxylation of 6-phosphogluconate to ribulose 5-phosphate and CO(2), with concomitant reduction of NADP to NADPH

### The expression of hub genes in human psoriasis samples

**Fig. 4:**
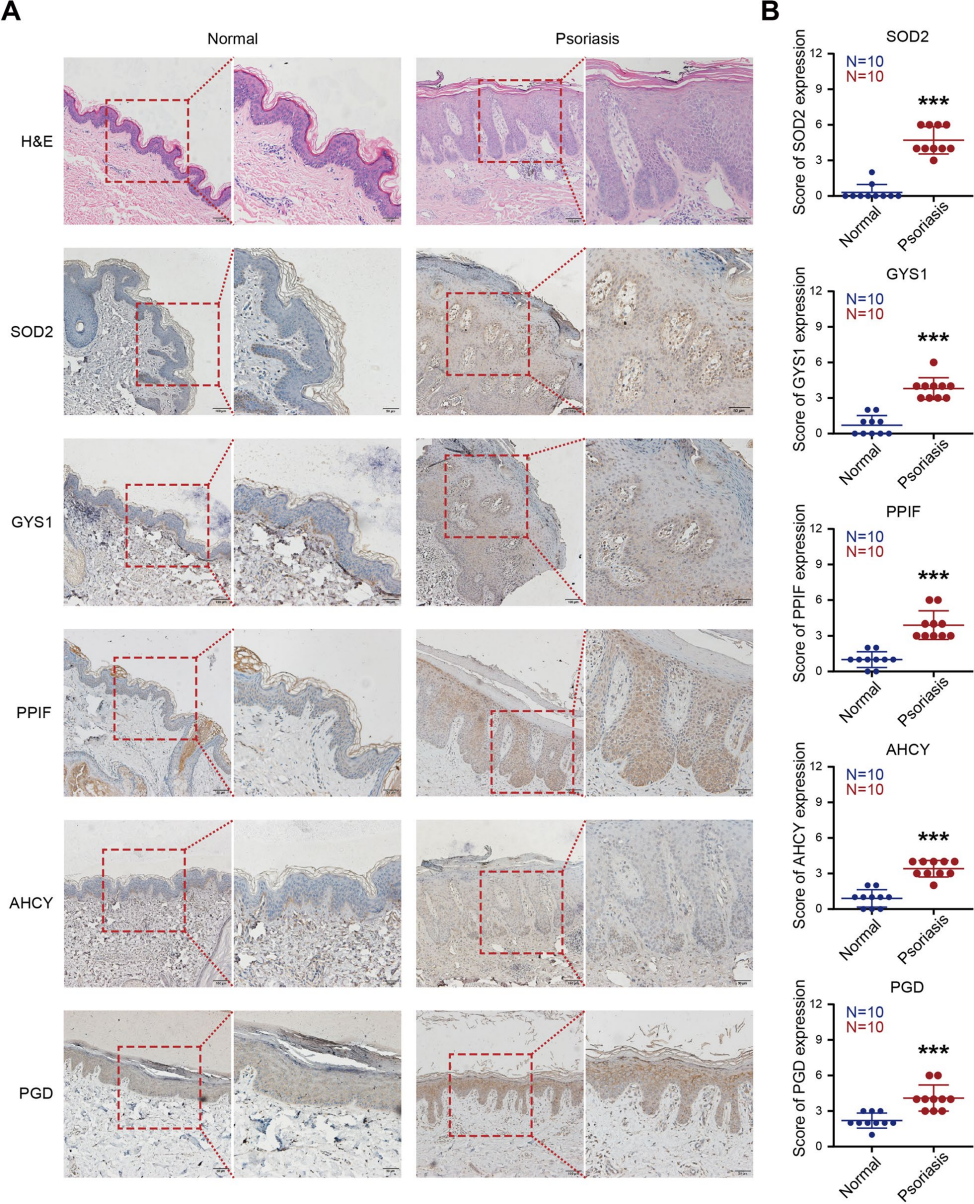
The expression of 5 hub genes in psoriasis samples by IHC

To validate the expression of 5 hub genes in psoriasis, we detected mRNA levels and protein levels of hub genes in patients with psoriasis and healthy donors by RT-qPCR and IHC, respectively. The mRNA levels of hub genes were elevated in psoriatic lesions compared with normal samples (Additional file 3: Fig. S2). HE staining and IHC results showed in Fig. 4 also revealed that hub genes were overexpressed in psoriatic lesions than normal tissues. Taken together, these results suggested the potential role of hub genes in the pathogenesis of psoriasis.

### Identification of novel immune subtypes of psoriasis

**Fig. 5:**
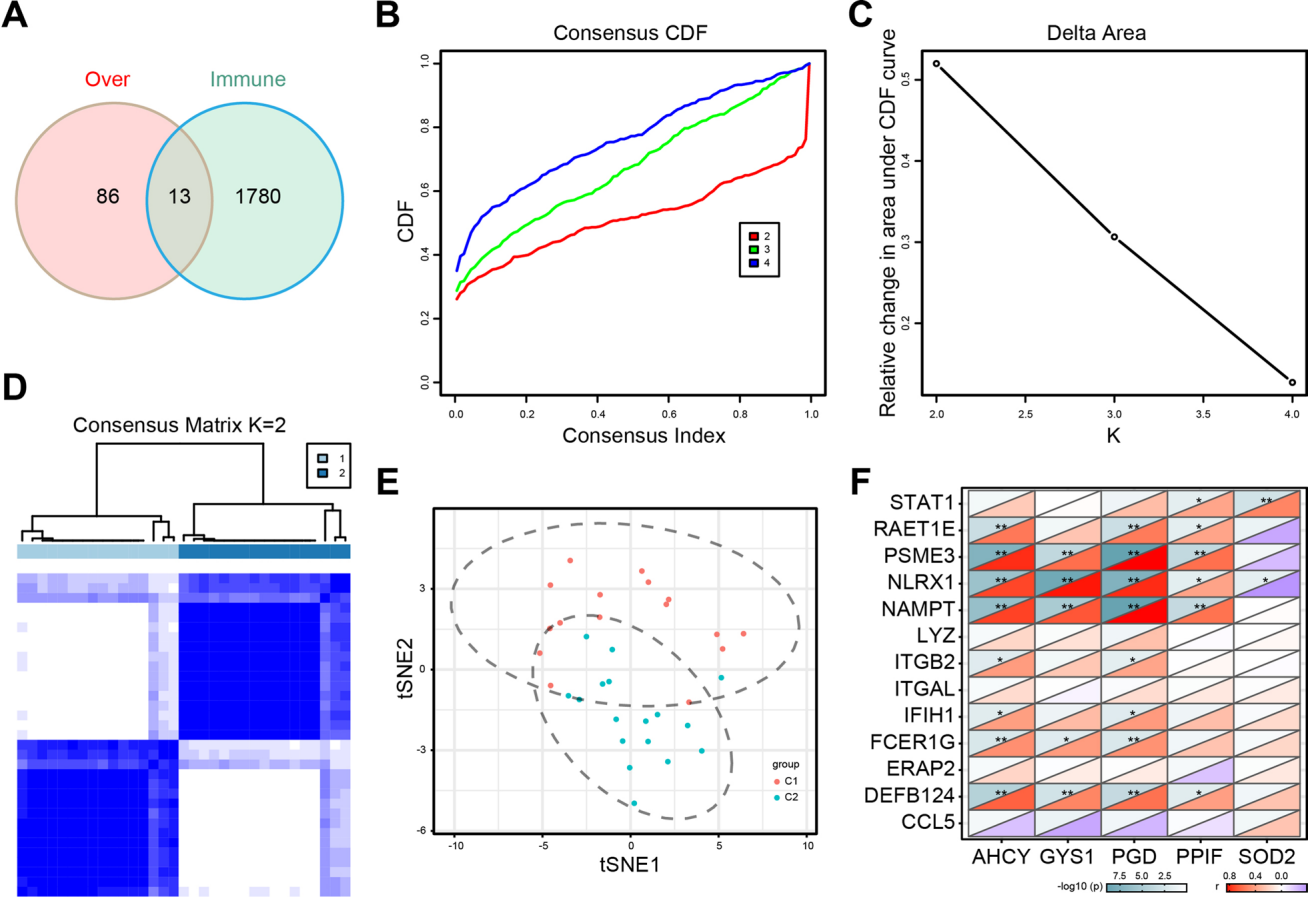
Potential immune subtypes of psoriasis. **A** Venn diagram identifying 13 genes that were shared by immune-related genes and up-regulated genes in psoriasis. **B**, **C** The cumulative distribution function curve (**B**) and δ area (**C**) of immune-related genes in GSE14905 cohort. **D** The heatmap of sample clustering. Consensus matrix for k = 2, which was the optimal cluster number. **E** The t-SNE plot for the data set. C1, colored by red. C2, colored by blue. **F** The heatmap between hub genes and two immune subtypes of psoriasis. ^*^P < 0.05; ^**^P < 0.01; ^***^P < 0.001

As we known, psoriasis is a common, relapsing and immune-related dermal disease. In order to provide effective therapeutic applications for the precise therapy of psoriasis, it is essential to recognize the immune status of patients with psoriasis by identifying novel immune subtypes. We firstly collected 1793 immune-related genes from IMMPORT database (https://www.immport.org/home). Then, 13 genes were obtained by intersection with 1793 immune-related genes and 99 up-regulated genes of psoriasis. (Fig. 5A). These 13 genes were further used to construct consensus clustering and identify immune subtypes of psoriasis. According to the cumulative distribution function and δ area, consensus matrix k = 2 was selected, where immune-related genes were in most stably clusters (Fig. 5B, C). Then, the immune subtypes of psoriasis were divided into two clusters and defined as C1 and C2 using ConsensusClusterPlus package (Fig. 5D), which were also demonstrated that the consensus clustering was significant by t-distributed Stochastic Neighbor Embedding (t-SNE) algorithm (Fig. 5E). Lastly, the association between 5 hub genes and 13 genes that used to identify immune subtypes of psoriasis was performed. The results found that there was a correlation between hub genes and immune subtypes of psoriasis (Fig. 5F).

### Immune infiltration analysis in different immune subtypes in psoriasis

**Fig. 6:**
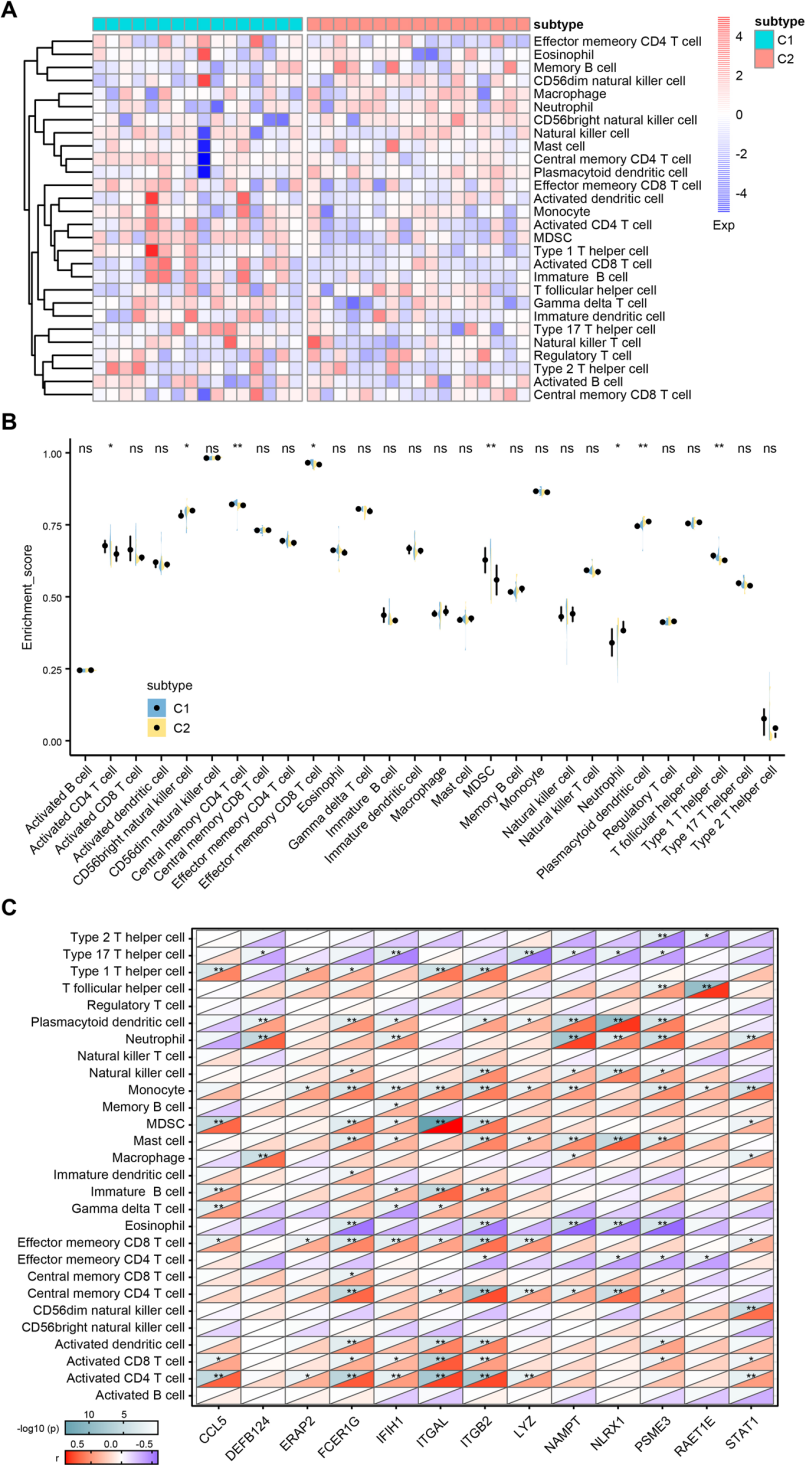
Analysis of immune infiltration of subtype C1 and C2. **A** Heatmaps of correlation between 28 immune infiltrating cells and different immune subtypes of psoriasis. Exp, Expression. **B** Differential enrichment scores of relevant immune cell signatures in C1 and C2 subtypes. **C** Heatmaps of correlation between 28 immune infiltrating cells and 13 genes

Analysis of immune infiltration in tissues is important to providing a guide and prediction for the treatments of psoriasis. In order to assess the immune infiltration profile among 2 subtypes of psoriasis, GSEA algorithm was conducted with 28 immune infiltrating cell markers from the public database (Fig. 6A). As is shown in Fig. 6B, there were considerable variations in the enrichment scores of several immune cells between C1 and C2. Specifically, C1 had higher enrichment scores of immune infiltration cells, such as activated CD4^+^ T cells, central memory CD4^+^ T cell, type 1 T helper cells, and Myeloid-derived suppressor cell (MDSC). However, the content of plasmacytoid dendritic cell, neutrophil and CD56^+^ natural killer cell was higher in C2. Additionally, the correlation was significant between 13 genes involved in immune subtypes of psoriasis and 28 immune infiltrating cells (Fig. 6C).

### Candidate drugs and mechanism of action for C1 and C2 subtypes of psoriasis

**Fig. 7:**
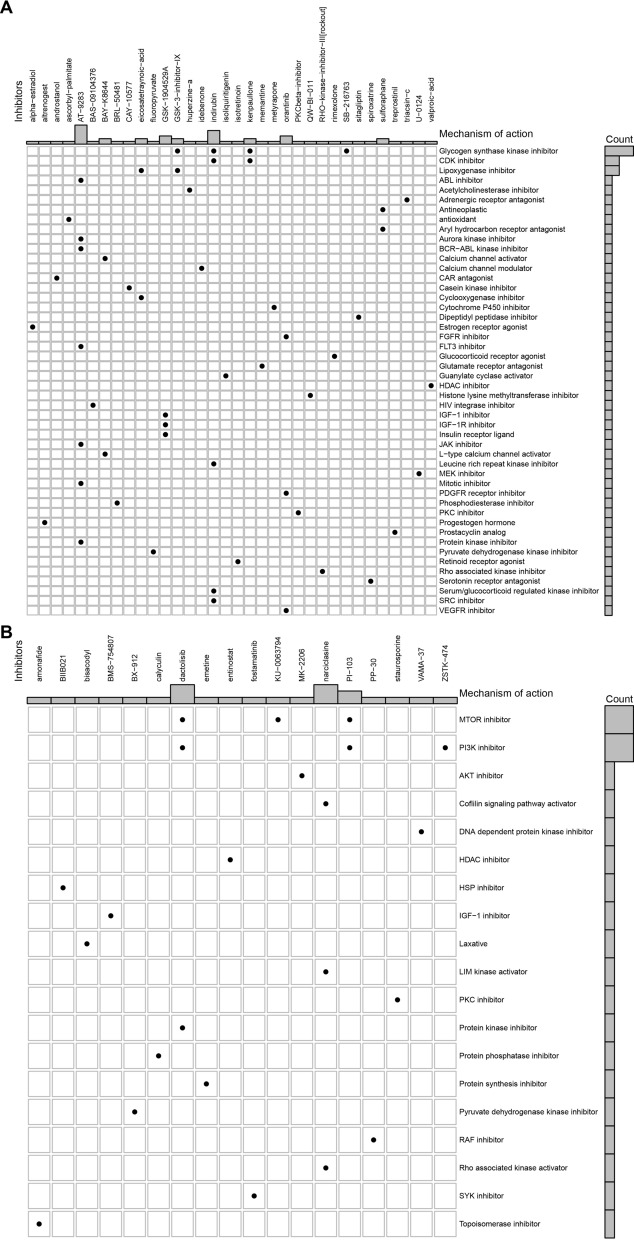
Candidate drugs and its mechanism for each subtype of psoriasis. **A**, **B** Connectivity Map analysis showed 48 molecular pathways targeted by 34 compounds in C1 (**A**) and 20 biological pathways targeted by 19 compounds in C2 (**B**)

As we known, the emergence of biologic therapy over the last two decades has shifted psoriasis management from treatment with conventional systemic treatments to those which target key cytokines in the inflammatory pathways involved in psoriasis. In order to investigate candidate drugs targeting biological pathways for different subtypes of psoriasis, Connectivity Map analysis was performed (https://clue.io/). Figure 7A revealed 48 molecular pathways targeted by 34 compounds in C1. Figure 7B represented 20 biological pathways targeted by 19 compounds in C2. However, there is no overlapping in drugs and mechanism of action between 2 subtypes, revealing that the psoriasis of different immune subtypes refers to different favorable therapy regimens. According to the most important mechanism of action for each immune subtype, there were 4 compounds involving the same mechanism of action of glycogen synthase kinase inhibitor in C1 (Fig. 7A). In terms of C2, there are 6 compounds involving the same mechanism of action of MTOR inhibitor and PI3K inhibitor (Fig. 7B), respectively.

## Discussion

More and more evidence suggested that it is necessary to identify potential biomarkers and targets for the precision therapy in skin inflammatory diseases [22, 23]. As an immune-related skin inflammatory diseases, the underlying etiology of psoriasis is not yet unclear. Evidence suggests the congregation of immune cells and their secreted inflammatory cytokines, leukocytes, and other inflammation-promoting factors in large amounts within the epidermal layers of the skin, driving an inflammatory milieu [24]. The treatment option for patient with psoriasis is based on the severity of the disease and decided by the clinicians with guidelines [25]. Nevertheless, patients still suffer from the recurrences of psoriasis and adverse effects of drugs [26–28]. Thus, it is urgent to provide potential biomarkers and selected targeted drugs for the precision therapy in psoriasis.

In our study, we first screened the DEGs from 33 lesioned psoriasis samples and 28 non-lesioned skin samples of patients based on GSE14905 from GEO database. To explore the functional enrichment in psoriasis, we then focused on the 99 up-regulated genes using GSEA and KEGG analysis. Figure 1A, B revealed that these genes were enriched in the natural killer cell mediated cytotoxicity, neutrophil extracellular trap formation, neutrophil degranulation and adaptive immune system. In DOSE analysis, we found these up-regulated genes involved in several immune related diseases, such as atopic dermatitis, pustulosis of palms and soles lymphoma, lymphoma and inflammatory dermatosis. However, we found that down-regulated genes are less involved in immune related diseases in DOSE analysis (Additional file 4: Fig. S3). Therefore, we focused on the up-regulated genes in the follow-up analysis.

In order to identify the core targets of psoriasis, we constructed PPI networks using Metascape database and studied the properties of these networks. We found that SOD2, PGD, PPIF, GYS1 and AHCY are five hub genes in PPI networks, indicating the potential role in the psoriasis initiation and progression. It is reported that SOD2 transforms toxic superoxide into hydrogen peroxide and diatomic oxygen, and its abundance in lesioned skin [18]. The elevated expression of SOD2 in skin lesions and the plasma activity of SOD were observed in patients with psoriasis that was assumed to be a protective response against oxidative stress. In terms of PGD, which involved in the pentose phosphate pathway, supports inflammation in atopic dermatitis lesions [20]. However, the role of other genes has not been explored in psoriasis. Therefore, the correlation of hub genes was valued by Pearson’s analysis. Further, the relations between 5 hub genes and immune infiltration were calculated, which showed that the levels of AHCY, PPIF and PGD were significantly correlated with the increased immune infiltrating cells, such as macrophages, activated CD8^+^ T cell and activated dendritic cell. As we known, many investigators have now demonstrated that multiple immune cell types are present in psoriasis, including dendritic cells, T cells and macrophages, which contribute to disease pathogenesis and drive keratinocyte proliferation [12].

To verify the expression of five hub genes in clinical psoriasis samples, both RT-qPCR and IHC found that hub genes were up-regulated in patients with psoriasis than healthy donors. Together, the 5 hub genes (SOD2, PGD, PPIF, GYS1 and AHCY) play pivotal roles in the development of psoriasis. The findings might help in identifying therapeutic targets and regimens in the treatment of psoriasis.

The immune characteristics of different models can provide a theoretical basis for classifying the immune subtypes of psoriasis. Therefore, immune subtypes of psoriasis were identified using ConsensusClusterPlus package based on the cumulative distribution function and δ area. According to the 13 genes that were shared by immune-related genes and up-regulated genes, the immune subtypes of psoriasis were divided into 2 clusters and defined as C1 and C2. The correlation between hub genes and two immune subtypes of psoriasis were further analyzed. In the immune infiltration assay, C1 had more enrichment in activated CD4^+^ T cell, central memory CD4^+^ T cell, type 1 T helper cell and MDSC. However, C2 had higher content of immune infiltration cells, such as plasmacytoid dendritic cell, neutrophil and CD56^+^ natural killer cell. It is reported that psoriasis is an inflammatory skin disease with strong neutrophil infiltration and high levels of the antimicrobial peptide [29]. Amount of antimicrobial peptides were released to induce psoriatic inflammatory lesions by stimulating plasmacytoid dendritic cells, suggesting an essential role of plasmacytoid dendritic cells in the pathogenesis of psoriasis [30, 31]. In terms of natural killer cell, it is observed decreased after treatment of psoriasis [32]. The transplantation of natural killer cell leads to the psoriasis induction [33]. Moreover, the correlation between 13 genes involved in immune subtypes of psoriasis and 28 infiltrating cells was demonstrated significant in our study. Further, Connectivity Map analysis was performed and found few connections in drugs and mechanisms between 2 subtypes, revealing that the psoriasis of different immune subtypes might have different favorable therapy regimens.

In conclusion, we selected 5 hub genes as potential diagnostic biomarkers through PPI networks and functional enrichments. Notably, 2 novel immune subtypes of psoriasis were identified. Further, the immune infiltration and candidate drugs for different subtypes were explored. Our findings might give insight into the pathogenesis of psoriasis and provide effective therapeutic applications for the precise treatment of psoriasis.

## Supplementary Information

**Additional file 1:** **Table S1.** List of RT-qPCR primers.

**Additional file 2:** **Figure S1.** Flow chart of analysis design.

**Additional file 3:** **Figure S2.** RT-qPCR analysis of hub genes in human normal tissues and psoriasis tissues. Data (A) are plotted as means ± SD from three independent measurements, *P < 0.05; **P < 0.01; ***P < 0.001, by unpaired two-tailed Student t test. The comparison of the hub genes levels in these two groups is analyzed in (B). *P < 0.05; **P < 0.01; ***P < 0.001, by unpaired two-tailed Student t test.

**Additional file 4:** **Figure S3.** The disease enrichment by DOSE analysis based on down-regulated genes of psoriasis.

## Data Availability

Datasets related to this article are from public database (GSE14905). All data generated or analyzed during this study are included in this article/Additional files.
