# Supplementary material for: Identification of novel immune subtypes and potential hub genes of patients with psoriasis
Source: J Transl Med. 2023 Mar 8;21:182. doi: 10.1186/s12967-023-03923-z (PMC9993638; doi:10.1186/s12967-023-03923-z)
Supplement: Supplementary file 1 — Additional file 1: Table S1. List of RT-qPCR primers. [file 12967_2023_3923_MOESM1_ESM.docx]

**Additional file 1: Table S1. List of qRT-PCR primers.**

| Gene | Forward primer (5'-3') | Reverse primer (5'-3') |
| --- | --- | --- |
| PPIF | GAAGGCAGATGTCGTCCCAAA | GGAAAGCGGCTTCCGTAGAT |
| SOD2 | GCTCCGGTTTTGGGGTATCTG | GCGTTGATGTGAGGTTCCAG |
| GSY1 | TTTATGGGCATCTGGACTTCAAC | CGCTGCCGTTCACTCTGAG |
| AHCY | TAGCAGGCTATGGTGATGTGG | ATGGGGTCAATCTCGGTGATG |
| PGD | ATGGCCCAAGCTGACATCG | AAAGCCGTGGTCATTCATGTT |
