# Supplementary figures and images for: Identification of novel immune subtypes and potential hub genes of patients with psoriasis
Source: J Transl Med. 2023 Mar 8;21:182. doi: 10.1186/s12967-023-03923-z (PMC9993638; doi:10.1186/s12967-023-03923-z)

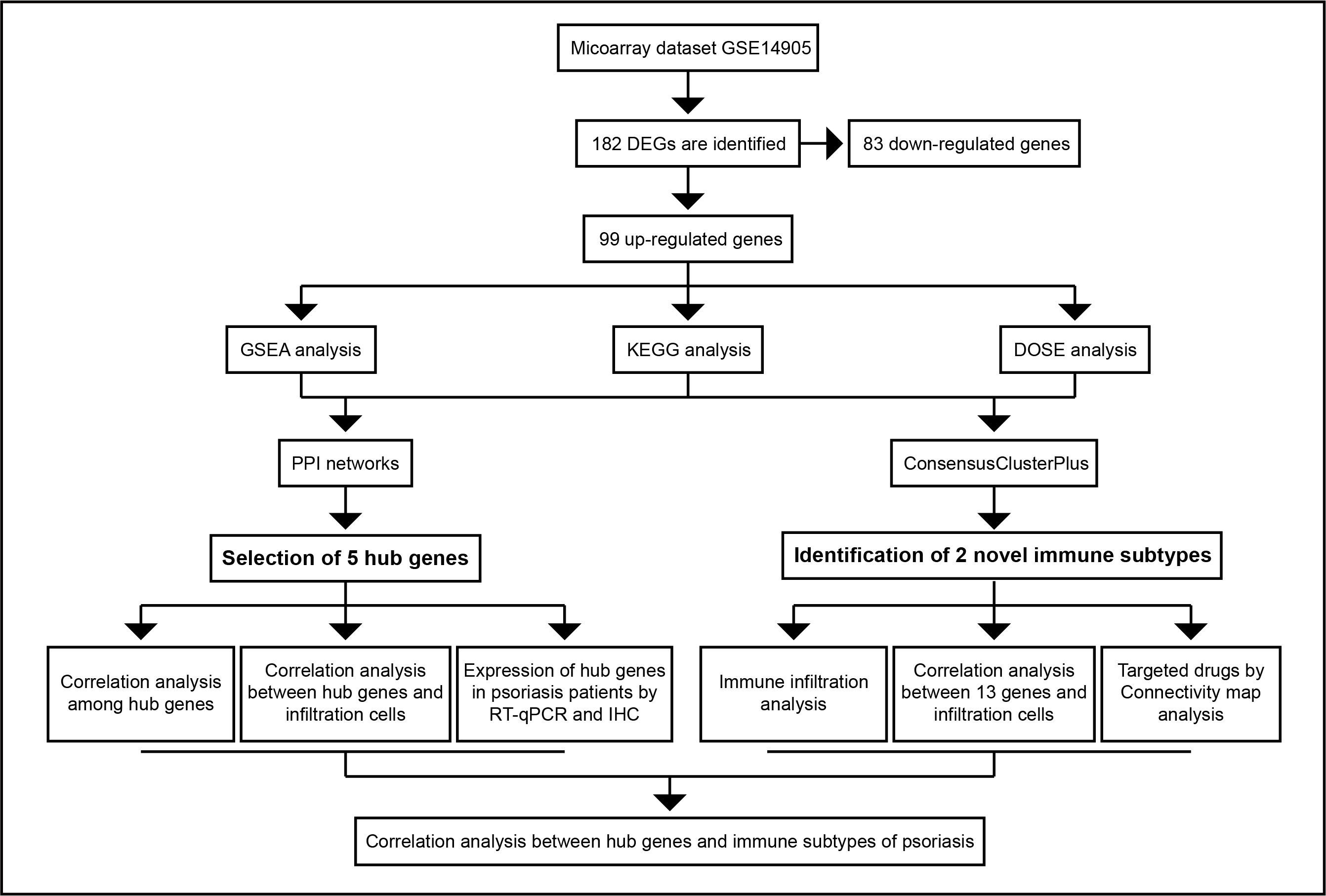

Supplement: Supplementary file 2 — Additional file 2: Figure S1. Flow chart of analysis design. [file 12967_2023_3923_MOESM2_ESM.jpg]

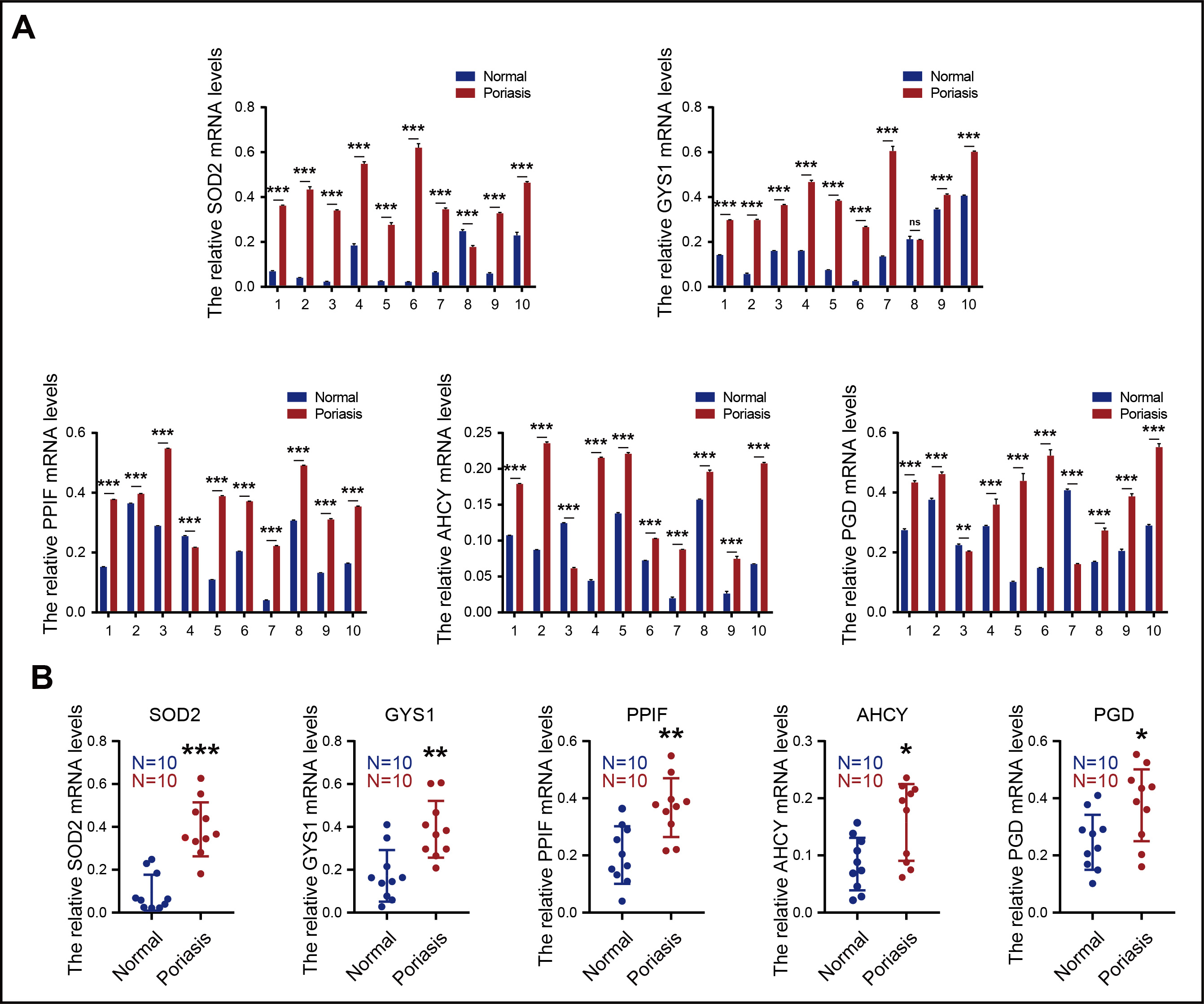

Supplement: Supplementary file 3 — Additional file 3: Figure S2. RT-qPCR analysis of hub genes in human normal tissues and psoriasis tissues. Data (A) are plotted as means ± SD from three independent measurements, *P < 0.05; **P < 0.01; ***P < 0.001, by unpaired two-tailed Student t test. The comparison of the hub genes levels in these two groups is analyzed in (B). *P < 0.05; **P < 0.01; ***P < 0.001, by unpaired two-tailed Student t test. [file 12967_2023_3923_MOESM3_ESM.jpg]

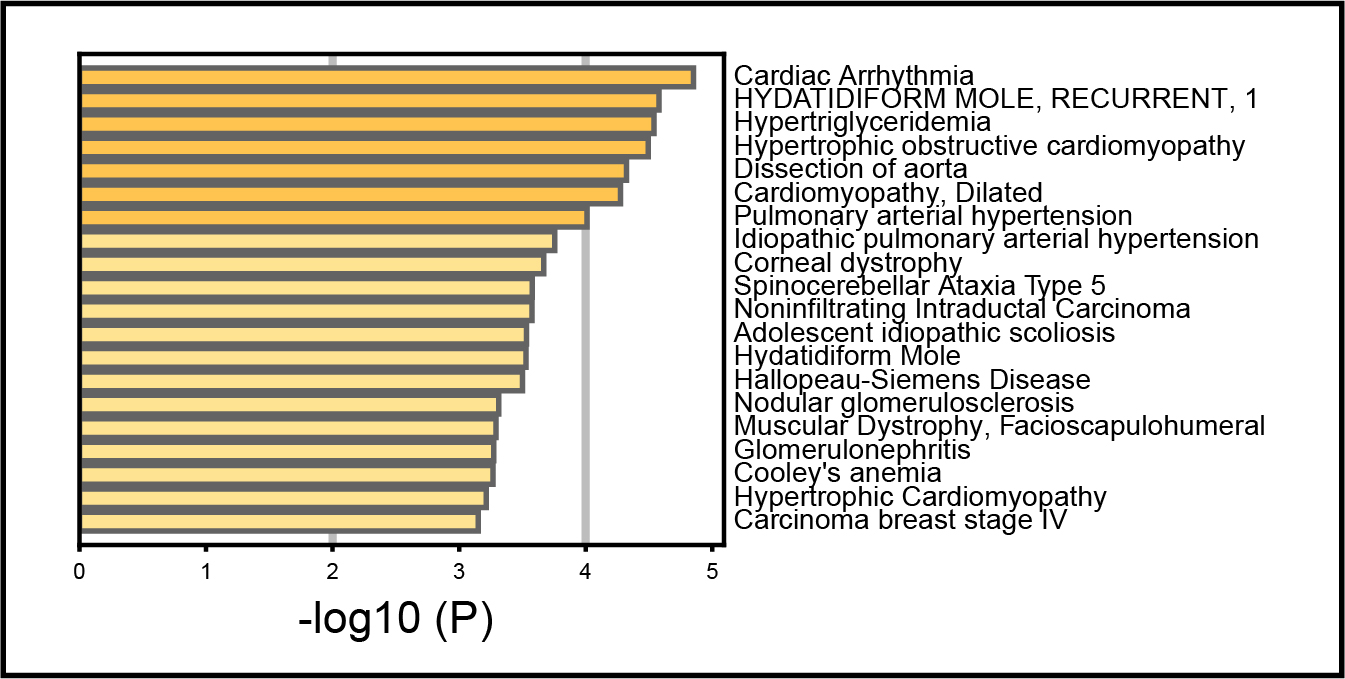

Supplement: Supplementary file 4 — Additional file 4: Figure S3. The disease enrichment by DOSE analysis based on down-regulated genes of psoriasis. [file 12967_2023_3923_MOESM4_ESM.jpg]
